# Trauma reminders and disgust: The roles of posttraumatic stress disorder symptom severity, trauma type, and reminder type

**DOI:** 10.1002/jts.23076

**Published:** 2024-07-18

**Authors:** M. Shae Nester, Blair E. Wisco

**Affiliations:** ^1^ Department of Psychology University of North Carolina at Greensboro Greensboro North Carolina USA

## Abstract

Disgust is a common emotional response to trauma but is studied less frequently than fear or other negative emotions. In laboratory settings, individuals with a history of sexual assault report more disgust following exposure to trauma reminders than those exposed to other trauma types, and people with more severe posttraumatic stress disorder (PTSD) symptoms typically report more disgust than those with lower symptom levels. It remains unknown whether this association is also present in ecological contexts and if these associations vary by trauma reminder type. The present sample included 80 trauma‐exposed community members (PTSD: *n* = 39, no PTSD: *n* = 41) who completed up to 17 prompts per day for 3 days (2,158 total completed surveys). Multilevel models indicated that trauma reminders were associated with increased feelings of disgust, *B* = 0.16, *SE* = 0.06, *p* < .001, which was consistent across trauma reminder types, *p* < .001–*p =* .001. PTSD symptom severity moderated the association between trauma reminders and disgust such that it was stronger for participants with higher CAPS‐5 scores, *B* = 0.02, *SE* = 0.01, *p* = .011. All trauma reminder types demonstrated the same pattern of moderation, *p*s = .003–.022, except flashbacks, *p* = .070. Trauma type was not a significant moderator of any trauma reminder type, *p*s = .193–.929. These findings suggest that trauma reminders encountered in daily life are associated with feelings of disgust. The results underscore the importance of exploring disgust as a trauma‐related emotional experience among trauma survivors.

Posttraumatic stress disorder (PTSD) was once conceptualized as an anxiety disorder predominantly characterized by emotions of fear, horror, and helplessness (Foa et al., 1989). However, the diagnosis was revised in the *Diagnostic and Statistical Manual of Mental Disorders* (5th ed.; *DSM‐5*; American Psychiatric Association [APA], 2013) based on findings that there are many emotional reactions that can occur in the wake of trauma (McLean & Foa, 2017). One updated criterion for PTSD is that of a “persistent negative emotional state,” which includes emotions such as fear, horror, anger, guilt, and shame, among others. This change reflects the range of emotional reactions an individual might experience following trauma exposure.

One common emotional reaction to trauma is disgust (Hathaway et al., 2010; Power & Fyvie, 2013). Disgust is conceptualized as a rejection or revulsion response meant to distance an individual from a potentially harmful or noxious encounter (Davey, 2011). In some cases, disgust can be an evolutionarily adaptive reaction that compels people to avoid potentially hazardous situations (Rozin & Fallon, 1987). However, sometimes disgust can be maladaptive such that people can experience persistent and intense repulsive reactions to stimuli that pose no threat or hazard (e.g., self‐disgust; Brake et al., 2017).

Traumatic events that include exposure to contaminants such as revulsive smells or bodily products (e.g., vomit, blood, semen) and situations involving disease, death, betrayal; violations of morality; or sexual violation are more likely to evoke feelings of disgust than events without these characteristics (e.g., de Silva & Marks, 1999; Fairbrother & Rachman, 2004; Steil et al., 2011). Aligned with behavioral models of PTSD, conditioning is one mechanism by which feelings of disgust can develop and persist following exposure to trauma (Badour, Feldner, Blumenthal, & Knapp, 2013). Intense feelings of disgust during a traumatic event may result in associations between previously neutral stimuli and aversive stimuli present during the traumatic event through the process of classical conditioning. In doing so, reminders of the trauma can elicit feelings of disgust even after the immediate threat of the traumatic event has ended. Conditioned disgust reactions can contribute to attempts to avoid internal (e.g., thoughts, memories, emotions) and external (e.g., people, places, situations) reminders of the trauma, demonstrating principles of operant conditioning. Persistent avoidance of trauma‐related and disgust‐inducing stimuli ultimately maintain PTSD symptoms and disgust reactions by preventing extinction learning (Badour, Feldner, Blumenthal, & Knapp, 2013).

Disgust has also been linked to evaluative conditioning wherein the emotional valence of a conditioned stimulus is transferred to a previously neutral stimulus due to its co‐occurrence (Baeyens et al., 1988; C. R. Jones et al., 2010). Evaluative conditioning refers to a relatively immediate evaluation of the stimulus (e.g., as good or bad, liking or disliking) and occurs in the absence of a prediction about the recurrence of the unconditioned stimulus, which distinguishes it from classical conditioning. For example, feelings of disgust may be transferred from disgust‐eliciting aspects of the traumatic event to other stimuli present, such as the self. In this case, people may view themselves as dirty, contaminated, or disgusting following the traumatic event. Although most emotions are vulnerable to both classical and evaluative conditioning, disgust is believed to be especially impacted by evaluative conditioning in the context of trauma (Badour & Feldner, 2018), and, in turn, is more resistant to extinction learning as compared to other emotional reactions (e.g., fear; Baeyens et al., 1988; Olatunji, Forsyth, & Cherian, 2007). Further, individuals often provide tautological reasons to justify their disgust (e.g., “I'm disgusted because he is gross”), whereas other emotions are often justified using logical reasoning (e.g., “I'm sad because he betrayed my trust”; Russel & Giner‐Sorolla, 2011), making different emotions sensitive to different types of treatment. There are several cognitive mechanisms by which disgust occurs, such as appraisals of disgust‐eliciting stimuli and information‐processing biases (Williams et al., 2009). Based on the research to date, PTSD‐related feelings of disgust may still respond to cognitive behavioral interventions (Jung & Steil, 2012, 2013; Steil et al., 2011).

Although many people experience peritraumatic disgust, a smaller proportion of individuals are expected to continue experiencing trauma‐related disgust over time (A. C. Jones et al., 2020). Approximately 10% of people with PTSD endorse disgust as their primary negative emotion following trauma (Power & Fyvie, 2013). Disgust reactions are often measured through evaluations of trait feelings of disgust, such as with the Disgust Propensity and Sensitivity Scale (Olatuniji, Cisler, et al., 2007), or general feelings of disgust, such as with a single‐item rating scale. Disgust propensity (i.e., the frequency or ease of experiencing disgust) and sensitivity (i.e., the degree of distress when experiencing disgust) are both associated with the risk of experiencing prolonged disgust reactions following trauma (as reviewed in Badour & Feldner, 2018; A. C. Jones et al., 2020). General feelings of disgust are highly associated with PTSD status and severity (A. C. Jones et al., 2020). Individuals with PTSD report more persistent and frequent trait feelings of disgust compared to trauma‐exposed populations without PTSD (Finucane et al., 2012; Power & Fyvie, 2013). Feelings of disgust are also associated with other trauma sequelae, including suicide (Brake et al., 2017), hazardous drinking (Sonnier et al., 2019), dissociation, and self‐harm (Bradley et al., 2019).

To study the association between trauma and disgust, researchers have employed laboratory‐based standardized (e.g., film clips, pictures of trauma‐related content) and idiographic (e.g., personalized script‐driven imagery) methods to understand whether trauma reminders elicit disgust. After engaging in trauma script–driven imagery, trauma survivors typically report elevated feelings of disgust compared to their baseline levels (e.g., Badour et al., 2011; Shin et al., 1999). Some studies have found that people with PTSD (Olatunji et al., 2009; Shin et al., 1999) or those with higher levels of PTSD symptom severity (Badour et al., 2011; Badour & Feldner, 2013) report higher feelings of disgust than those with fewer PTSD symptoms in response to script‐driven imagery. Of note, other studies have failed to replicate differences in disgust ratings based on PTSD status or symptom severity (e.g., Ojserkis et al., 2014; Orr et al., 1993).

Although survivors of many types of trauma endorse feelings of disgust (e.g., Engelhard et al., 2011; Feldner et al., 2010), sexual violence may be particularly highly associated with disgust. Sexual trauma typically involves betrayal, violations of morality, sexual violation, and bodily fluids, each of which is known to evoke feelings of disgust (e.g., de Silva & Marks, 1999; Gershuny et al., 2003). Interestingly, childhood sexual abuse survivors report feelings of disgust even more frequently than emotions like anger, sadness, and fear (Coyle et al., 2014). Individuals with a history of sexual assault also demonstrate heightened disgust reactivity in response to trauma imagery (Badour et al., 2011; Badour, Feldner, Babson et al., 2013). Sexual violence is often associated with disgust and PTSD through feelings of mental contamination and dirtiness (Badour, Feldner, Babson, et al., 2013; Brake et al., 2021; Fairbrother & Rachman, 2004). Taken together, these studies suggest that sexual violence may be especially associated with feelings of disgust as compared to other trauma types.

Thus, there is preliminary support for the association among disgust, trauma, and PTSD (Badour & Feldner, 2018). After being exposed to laboratory‐based trauma reminders, survivors of trauma have been shown to endorse feelings of disgust (e.g., Badour et al., 2011). Individuals with PTSD and higher levels of PTSD symptom severity (e.g., Olatunji et al., 2009), and those who have experienced sexual violence (e.g., Badour & Feldner, 2013), demonstrate even higher trait and laboratory‐elicited feelings of disgust. These effects have not been consistently replicated, however, highlighting the need for continued exploration of these constructs. A significant methodological limitation is that most studies employ laboratory‐based external reminders of the trauma, which may not necessarily reflect the variety of trauma reminders that survivors encounter in daily life (e.g., flashbacks, trauma thoughts, intrusive memories). Indeed, it is not a common experience for trauma survivors to hear a rewritten account of their traumatic event read to them in a laboratory setting (i.e., lab). Therefore, it remains unclear whether trauma reminders encountered in daily life similarly elicit disgust and whether some types of trauma reminders (e.g., flashbacks) cooccur with stronger feelings of disgust than others (e.g., external reminders).

Ecological momentary assessment (EMA) is an advantageous method for studying disgust and trauma reminders because it allows for the repeated collection of data in naturalistic settings. By doing so, a broader range of trauma reminders encountered in daily life (e.g., thoughts about the trauma, unwanted memories, flashbacks, external reminders) can be considered, which is a more comprehensive and ecologically valid measure of trauma reminders than laboratory‐based script‐driven imagery. Further, EMA enables participants to report both the occurrence and intensity of disgust as it occurs in daily life, which allows for moment‐to‐moment fluctuations in emotional experiences to be captured. Despite the apparent advantages of EMA, one drawback is that the survey prompts may interrupt participants’ naturalistic daily living experiences, which, in turn, may impact the ecological validity of the study and resultant findings.

The present study used EMA to explore the association between trauma reminders and feelings of disgust while considering the role of PTSD symptom severity, trauma type (i.e., sexual assault vs. non–sexual assault as the index traumatic event), and trauma reminder type (i.e., trauma thoughts, intrusive memories, flashbacks, and external reminders). We made five hypotheses. First, disgust ratings were expected to be higher during moments when trauma reminders were present. Second, we expected that both Clinician‐Administered PTSD Scale for *DSM‐5* (CAPS‐5; Weathers et al., 2013a) score would be associated with disgust ratings on average such that CAPS‐5 score would be positively associated with disgust. Third, we posited that participants who had been exposed to sexual trauma would experience more disgust, on average, than those exposed to nonsexual trauma types. Fourth, the association between trauma reminders and disgust was predicted to be stronger for individuals with higher CAPS‐5 scores. Finally, we hypothesized that trauma type would impact the relation between trauma reminders and feelings of disgust such that the association between trauma reminders and disgust would be stronger for individuals who endorsed sexual assault as their index traumatic event compared to those who endorsed other types of index traumatic events. An exploratory aim of the study was to examine whether any of the effects discussed varied by trauma reminder type (i.e., intrusive memories, trauma thoughts, flashbacks, or external reminders).

## METHOD

### Participants

This is a secondary analysis of a dataset that included 80 trauma‐exposed community members who completed a larger study (i.e., the Ambulatory Physiological Assessment of PTSD study; Wisco et al., 2024). Participants were an average age of 21.79 years old (*SD* = 4.21). Most participants were women (*n* = 60, 75.0%) and White (*n* = 28, 35.0%) or Black (*n* = 23, 28.8%). Based on the CAPS‐5, 39 participants met the criteria for current (i.e., past‐month) PTSD, and 41 participants did not meet the criteria for current PTSD. See Supplementary Table S1 for an overview of all demographic information.

Participants were eligible if they were between 18 and 40 years of age, had a body mass index between 18.5 and 34.9 kg/m^2^, spoke English, and reported experiencing at least one *DSM‐5* Criterion A traumatic event. Exclusion criteria for the study were current pregnancy, a history of cardiovascular disease, medications known to affect cardiovascular functioning (e.g., antidepressants), psychosis, past‐month trauma exposure, and endorsing dissociative symptoms on a prescreening measure. Exclusion criteria were based on the aims of the parent study, which included psychophysiological measures not analyzed here.

### Procedure

The present study includes secondary analyses from a parent study focused on understanding psychophysiological reactivity to trauma reminders encountered in daily life (Wisco et al., 2024). Participants were recruited using community flyer postings and online advertisements, as well as from a psychology clinic and a repository of trauma‐exposed adults who completed prior studies in the lab and consented to future contact. After providing informed consent, people interested in the study completed online prescreening questionnaires, which included questions about lifetime trauma history, past‐month PTSD symptoms, psychosis, medications and health conditions known to affect physiological data, and height and weight. Participants with and without PTSD were recruited and matched based on trauma type, age, race and ethnicity, and BMI.

Participants who were invited into the study then completed two lab sessions and three days of ambulatory assessment (i.e., EMA paired with ambulatory physiological monitoring). In the first lab session, participants completed structured clinical interviews, including the CAPS‐5, and other questionnaires that are not analyzed here. Within approximately 1 week of the first lab session (*M* = 5.48 days until completion of the first ambulatory session, *SD* = 4.50), participants completed 3 days of ambulatory assessment. On the first morning of ambulatory assessment, participants came into the lab to be connected to the mobile physiological device and were oriented to the EMA questionnaires, which were completed on a Qualtrics Offline survey platform on a Lenovo tablet. Participants returned to the lab on the morning of each ambulatory assessment to be connected to an ambulatory device and receive a tablet for EMA.

EMA prompts were delivered up to 17 times per day between 10 a.m. and 10 p.m. Prompts were pseudorandomized such that they were separated by a randomized time interval between 30 and 90 min (*M* = 53.67 min, *SD* = 13.34, range: 31–73 min). The tablet alarm alerted participants to complete each observation, after which participants had 20 min to respond. At each signal prompt, participants were asked to complete the EMA questionnaire based on their experience over the previous 10 min.

At the final lab session, participants completed additional self‐report questionnaires and a script‐driven imagery procedure and were provided monetary compensation for their participation. In the present study, only the prescreening questionnaire (Life Events Checklist for *DSM‐5*; LEC‐5; Weathers et al., 2013b), first lab session clinical interview (CAPS‐5), and EMA items about trauma reminders and feelings of disgust were analyzed. All study procedures were approved by the University of North Carolina at Greensboro Institutional Review Board and complied with ethical standards set forth by the American Psychological Association.

### Measures

#### Index trauma type

The LEC‐5 (Weathers et al., 2013b) was used to assess lifetime history of trauma exposure and index trauma type. The LEC‐5 assesses 17 different types of traumatic events and has demonstrated adequate convergent validity but considerable variability in its temporal stability (Gray et al., 2004; Pugach et al., 2021). Notably, the measure has shown reliable test–retest agreement for endorsing sexual assault, in particular (Pugach et al., 2021). If participants endorsed more than one type of event, they were prompted to nominate one event as the “worst” (i.e., index traumatic event). The self‐nominated index trauma was used to dichotomize participants by trauma type (i.e., sexual assault vs. non–sexual assault). A total of 38.8% of participants (*n* = 31) reported sexual assault as their index traumatic event, and the remaining 61.2% (*n* = 49) reported non–sexual assault index traumatic events, such as physical assault, natural disasters, serious illness or injuries, combat, or exposure to sudden or accidental death. See Supplementary Table S1 for an overview of reported index traumatic events.

#### PTSD symptom severity

PTSD symptom severity was measured using the CAPS‐5 (Weathers et al., 2013a). The CAPS‐5 is a structured clinical interview used to assess the 20 core *DSM‐5* symptoms of PTSD as well as depersonalization and derealization. Items are rated on a 5‐point Likert scale ranging from 0 (*absent*) to 4 (*extreme/incapacitating*). Items from the 20 core PTSD symptoms were totaled to provide an index of PTSD symptom severity. The CAPS‐5 has demonstrated strong psychometric properties, including internal consistency, interrater reliability, test–retest reliability, convergent validity, and discriminant validity (Weathers et al., 2018). All interviews were conducted by a trained graduate student, audio‐recorded, and independently scored by a second trained graduate student. Scoring discrepancies were resolved via consensus by the senior author, a doctoral psychologist with a specialization in PTSD. Interrater reliability on the CAPS‐5 was excellent, intraclass correlation coefficient (ICC) = .99.

#### Trauma reminders

Using EMA, participants were randomly prompted up to 17 times a day to report their exposure to trauma reminders in their daily lives. At each observation, the item stem “In the past 10 minutes…” preceded each of the following questions: (a) “I was thinking about the trauma (i.e., trauma thoughts),” (b) “I had unwanted memories of the trauma (i.e., intrusive memories),” (c) “I relived the trauma as though it were actually happening again (i.e., flashbacks),” and (d) “Something reminded me of the trauma (i.e., external reminders).” These prompts were operationalized to reflect four separate trauma reminder types: trauma thoughts, intrusive memories, flashbacks, and external reminders. Participants rated each item on a scale of 1 (*not at all*) to 7 (*very much*); no intermediate anchors were provided for scores of 2–6. If participants endorsed a scale option of two or higher on any of the four trauma reminder types, the respective trauma reminder type was coded as present (1); scores of 1 were coded as not present (0). An additional trauma reminder composite was calculated to capture whether any trauma reminders were present at a given prompt regardless of trauma reminder type (also coded as 1 = any reminder type present or 0 = no reminder type present).

#### Disgust

At each EMA observation, participants rated the extent to which they felt the following emotions in the prior 10 min: disgusted, afraid, worried, horrified, helpless, guilty, numb, ashamed, sad, angry, happy, relaxed, and safe. Items were rated on a 7‐point Likert scale ranging from 1 (*not at all*) to 7 (*very much*). Only disgust ratings were used in the present study. Continuous disgust ratings were used as the outcome variable for all analyses. For descriptive purposes, disgust was considered present if the participant endorsed a score of 2 or higher on any disgust item.

### Data analysis

Data were analyzed using multilevel modeling due to the nested (i.e., repeated observations nested within participants) nature of the data. Descriptive statistics were calculated using SPSS (Version 28), and multilevel models were conducted using M*plus* (Version 8.10; Muthén & Muthén, 2017). All analyses were estimated using maximum likelihood with robust standard errors, which is equipped to model nonnormally distributed outcome data (e.g., Savalei, 2010). Using disgust as the outcome, an unconditional model was estimated to determine the proportion of variance explained at the between‐person level, as indicated by the ICC. Next, three separate models were run to test the effects of trauma reminders (composite score), PTSD symptom severity, and trauma type on disgust. To test our first hypothesis, the first model included only trauma reminders as a Level 1 variable to assess the relation between trauma reminders and feelings of disgust. To test our second and third hypotheses, the second model included CAPS‐5 score, and the third model included trauma type (sexual vs. nonsexual) as Level 2 (person‐level) variables to assess differences in mean levels of disgust between groups.

Lastly, to test our fourth and fifth hypotheses, the full model was run, which included composite trauma reminder score at Level 1 and CAPS‐5 score (grand‐mean centered) and trauma type at Level 2. The variables were entered as predictors of both the intercept and slope, as demonstrated below:

Level 1:
$$\mathrm{Disgus}t_{ij}=\;\beta_{0j}+\beta_{1j}\left(\mathrm{Trauma}\;\mathrm{Reminder}\right)+r_{ij}$$
Level 2:
$$\begin{array}{ccc} \beta_{0j} & = & \gamma_{00}+\;\gamma_{01}\left(\mathrm{CAPS}-5\right)+\gamma_{02}\left(\mathrm{Trauma}\;\mathrm{Type}\right)\\ & & +\;u_{0j} \end{array}$$
$$\begin{array}{ccc} \beta_{1j} & = & \gamma_{10}+\;\gamma_{11}\left(\mathrm{CAPS}-5\right)+\gamma_{12}\left(\mathrm{Trauma}\;\mathrm{Type}\right)\\ & & +\;u_{0j} \end{array}$$

The full model was then repeated to examine each trauma reminder type separately.

## RESULTS

### Participant and EMA characteristics

Participants completed a total of 2,158 EMA surveys, which equates to an average of 26.98 surveys per participant. Disgust was endorsed in 6.1% (*n* = 131) of the surveys. Approximately 50% (*n* = 40) of participants endorsed feelings of disgust at least one time in the study. Among individuals who reported disgust, the mean disgust rating was 1.26 (*SD* = 0.85, range: 1–7), and these participants reported experiencing disgust in 11.3% of completed surveys. As shown in Supplementary Table S1, participants with and without a sexual assault index trauma type did not differ in PTSD symptom severity, *p* = .069; PTSD status, *p* = .185; age, *p* = .889; gender, *p* = .145; or race, *p* = .185. See Supplementary Table S2 for the correlations between the primary study variables.

### Disgust results

**FIGURE 1:**
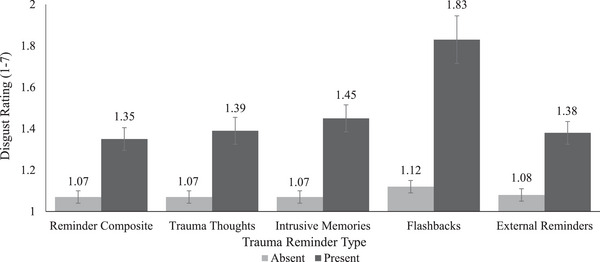
Disgust ratings in the absence and presence of trauma reminders. *Note*: Error bars show standard errors.

An unconditional model that included only disgust indicated the average disgust rating across all participants was 1.16, 95% confidence interval (CI) [1.08, 1.24], Akaike information criterion = 3,653.85, Bayesian information criterion = 3,670.88. Approximately 29.3% of the variability in disgust ratings could be attributed to differences between participants, ICC = .29. We then ran separate models for the trauma reminder composite and each trauma reminder type as Level 1 predictors. As hypothesized, there was a significant effect of trauma reminders on disgust for every trauma reminder type (see Figure 1) such that disgust ratings were higher when trauma reminders were present, *p*s < .001. The largest increase in disgust was endorsed during flashbacks, *B* = 0.71, *SD* = 0.20.

**FIGURE 2:**
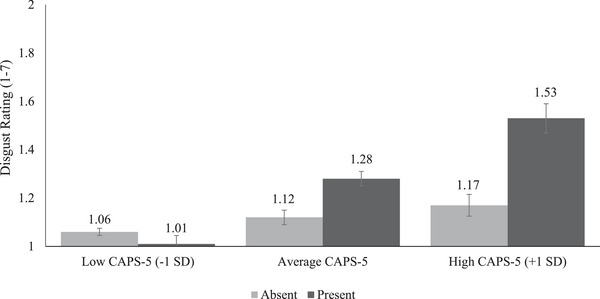
Disgust ratings in the absence and presence of trauma reminders, by posttraumatic stress disorder (PTSD) symptom severity. *Note*: Error bars show standard errors. The same pattern of results was demonstrated across trauma thoughts, intrusive memories, and external reminders. CAPS‐5 = Clinician‐Administered PTSD Scale for *DSM‐5*.

We next examined the effects of CAPS‐5 score and trauma type on disgust with separate means as outcomes models. CAPS‐5 score was significantly associated with mean disgust ratings, *B* = 0.01, *SE* = 0.004, *p* = 004, such that higher CAPS‐5 scores were associated with higher disgust, supporting our second hypothesis. In contrast, trauma type was not associated with mean disgust ratings, *B* = −0.06, *SE* = −0.08, *p* = .448, which did not support our third hypothesis. We then ran the full model including our three predictors with all possible cross‐level interactions, first with the composite score for trauma reminders, then separately for each trauma reminder type. CAPS‐5 score significantly predicted the slope of each trauma reminder type, *p*s = .003–.022, except flashbacks, *p* = .070, such that the association between trauma reminders and disgust became stronger as CAPS‐5 score increased, offering partial support for our fourth hypothesis (see Figure 2). However, contrary to our fifth hypothesis, trauma type did not significantly predict the slope for any trauma reminder type, *p*s = .193–.929. See Table 1 for all multilevel modeling results and Supplementary Table S3 for fit indices.

**TABLE 1:** Multilevel modeling results

	Reminder composite			Trauma thoughts			Intrusive memories			Flashbacks			External reminders		
	(*N* = 601^a^, 27.9%)			(*n* = 465^a^, 21.6%)			(*n* = 335^a^, 15.5%)			(*n* = 92^a^, 4.3%)			(*n* = 466^a^, 21.6%)		
Variable	*B*	*SE*	*p*	*B*	*SE*	*p*	*B*	*SE*	*p*	*B*	*SE*	*p*	*B*	*SE*	*p*
Fixed effects															
Intercept (g_00_)	1.12	0.06	< .001	1.12	0.05	< .001	1.12	0.05	< .001	1.15	0.05	< .001	1.10	0.05	< .001
PTSD symptoms (g_01_)	0.01	< .001	.096	0.01	< .001	.071	0.01	< .001	.046	0.01	< .001	.023	< .001	< .001	.095
Trauma type (g_02_)	−0.09	0.07	.179	−0.09	0.06	.126	−0.08	0.06	.212	−0.07	0.07	.281	−0.06	0.06	.350
Reminders (g_10_)	0.16	0.06	< .001	0.14	0.07	.033	0.25	0.09	< .001	0.55	0.22	.013	0.22	0.07	.001
PTSD x Reminders (g_11_)	0.02	0.01	.011	0.02	0.01	.022	0.03	0.01	.022	0.03	0.02	.070	0.02	0.01	.003
Trauma Type x Reminders (g_12_)	0.16	0.14	.254	0.25	0.19	.193	−0.02	0.20	.929	−0.07	0.39	.850	0.01	0.14	.923
Random effects															
Intercept (μ_0_)	0.03	0.03	.400	0.03	0.03	.377	0.03	0.03	.270	0.07	0.04	.064	0.02	0.03	.409
Slope (μ_1_)	0.24	0.08	.005	0.39	0.15	.009	0.36	0.12	.003	0.96	0.36	.007	0.24	0.08	.004
Level 1 (*r*)	0.25	0.06	< .001	0.24	0.06	< .001	0.24	0.06	< .001	0.25	0.07	< .001	.26	0.07	< .001
ICC	.27			.26			.25			.24			.26		
Fit indices															
AIC	3,382.93			3,272.31			3,301.22			3,365.48			3,419.10		
BIC	3,434.02			3,323.40			3,352.31			3,416.57			3,470.19		

*Note*: ICC = intraclass correlation coefficient; AIC = Akaike information criterion; BIC = Bayes information criterion.

^a^ Value represents how often the trauma reminder was endorsed across all ecological momentary assessment surveys.

### Robustness analyses

Analyses were also conducted by PTSD diagnostic status rather than a continuous CAPS‐5 score. PTSD status significantly predicted the slope of the trauma reminder composite, *B* = 0.27, *SD* = 0.13, *p* = .033; intrusive memories, *B* = 0.46, *SD* = 0.16, *p* = .003; and external reminders, *B* = 0.43, *SD* = 0.11, *p* < .001, but not other trauma reminder types, *p*s = .219–.481. Analyses were repeated using any history of sexual assault rather than including only participants who reported sexual assault as their index traumatic event. There were no changes in the pattern of results. Thus, index sexual assault was retained in primary analyses. All analyses were repeated excluding the eight individuals who reported dissociation on the CAPS‐5. There were no changes in the pattern of results, hence dissociative individuals were retained in the sample.

## DISCUSSION

Using EMA, the present study examined the association between trauma reminders and feelings of disgust among trauma‐exposed community members. Although feelings of disgust were relatively infrequent in the sample, they were endorsed at higher levels when trauma reminders were present and were associated with PTSD symptoms. As PTSD symptom severity increased, so did feelings of disgust in response to all trauma reminder types except for flashbacks, although the effect for flashbacks was in the same direction albeit nonsignificant. The largest increase in disgust was observed when flashbacks were present compared to other kinds of trauma reminders. Meanwhile, index trauma type (i.e., sexual vs. nonsexual) was not associated with disgust and did not moderate the association between trauma reminders and disgust. Overall, the findings suggest that trauma reminders encountered in daily life are associated with feelings of disgust and that PTSD symptoms, but not trauma type, are most commonly associated with the degree to which disgust is experienced.

Disgust was higher when trauma reminders were present, as predicted. In previous work, feelings of disgust have been elicited using trauma script‐driven imagery or distressing film paradigms meant to mimic trauma reminders (e.g., Badour & Feldner, 2018; A. C. Jones et al., 2020). Although these paradigms successfully elicited disgust reactions, the controlled lab setting and forced imaginal exposure did not necessarily reflect the nature of trauma reminders people experience in daily living. The present study measured trauma‐related thoughts, intrusive memories, flashbacks, and external reminders of the traumatic event as they naturally occurred in people's lives. Taken together, the present study extends past research by providing ecological support that “real‐world” trauma reminders are associated with feelings of disgust.

Notably, the largest increase in disgust was associated with flashbacks. Flashbacks are often highly somatic in nature and include reenactments of traumatic events, which may entail vivid recollections of disgust‐inducing stimuli (Brewin, 2015). Further, flashbacks most commonly occur among individuals with severe PTSD (Duke et al., 2008). Thus, these sensory, reliving, and severity components may explain why flashbacks are especially associated with disgust.

Although one of the study's strengths is that it captured these different types of trauma reminders in daily life, there are elements of the EMA study design that may have inherently impacted the study findings. For example, it might be argued that trauma‐related EMA prompts are external trauma reminders themselves. In turn, this could have been associated with trauma‐related experiences and reminders that might not have occurred as part of participants’ daily living experience had the EMA prompts not interrupted their naturalistic living. Thus, it is critical to acknowledge both the strengths and limitations of laboratory‐based and EMA designs, as methodological triangulation may be an avenue for drawing strong support—or lack thereof—in the study of trauma and disgust.

As expected, PTSD symptom severity was associated with disgust ratings and trauma reminders. This finding is aligned with past work that demonstrates that people with PTSD report more feelings of disgust both generally (Finucane et al., 2012; Power & Fyvie, 2013) and after being exposed to trauma reminders (Badour et al., 2011; Badour & Feldner, 2013; Engelhard et al., 2011; Shin et al., 1999). Indeed, feelings of disgust may be one negative emotional state that fits within conceptualizations of *DSM‐5* Criterion D4 (i.e., persistent negative emotional states; APA, 2013). Notably, Criterion D4 does not currently include disgust among the examples of specific negative emotions that may be present, listing instead fear, horror, anger, guilt, and/or shame. Our findings, coupled with other research on the role of disgust in PTSD, suggest that future research could examine whether disgust presents alongside other negative emotions to contribute to that symptom presentation.

PTSD symptom severity moderated the effect of most trauma reminders on disgust levels such that the association between trauma reminders and disgust was stronger for participants with higher levels of PTSD symptom severity. Individuals with more severe PTSD symptoms typically demonstrate more emotional reactivity to trauma reminders, partially due to conditioning that maintains associations between painful emotions and trauma‐related stimuli (Badour et al., 2012), which explains the heightened disgust that co‐occurs with trauma reminders. One of the aims of PTSD treatment is to promote the extinction of emotional responses to trauma‐related cues (Bryant, 2019). With increased desensitization to trauma cues comes less distress and reactivity to trauma reminders, theoretically resulting in lower PTSD symptom levels and fewer trauma reminder–related feelings of disgust. Disgust can be resistant to change or slow to improve with traditional exposure and extinction attempts, namely for obsessive–compulsive disorder–related disgust (McKay, 2006), specific phobia–related disgust (Smits et al., 2002), and disgust related to others (Olatunji, Forsyth, et al., 2007). To date, it appears that PTSD‐related disgust is amendable with exposure‐based treatments for trauma in that disgust reactions decrease along with PTSD symptoms (Jung & Steil, 2012, 2013; Steil et al., 2011). Future work is needed to understand the role of disgust in PTSD treatment and improve treatment options for disgust‐related psychopathology.

Although PTSD symptoms were associated with disgust and trauma reminders, trauma type was not. These findings contradict prior research suggesting that people with a history of sexual trauma experience more disgust both on average and in response to trauma reminders relative to survivors of other kinds of trauma (Badour, Feldner, Babson, et al., 2013; Brake et al., 2021; Coyle et al., 2014; Feldner et al., 2010). The present study did not measure contamination, such as mental contamination or physical contamination experienced at the time of the event (e.g., bodily fluids present during physical assault or accident), or disgust‐related factors, such as disgust sensitivity or propensity. It is possible that these other constructs, which are closely associated with posttraumatic disgust (as reviewed by Badour & Feldner, 2018; A. C. Jones et al., 2020), are mechanisms that are unaccounted for but explain feelings of disgust among people with a history of sexual trauma above and beyond the presence of the index sexual trauma itself.

Several limitations to the present study must be considered along with future directions for study. First, floor effects may have occurred with disgust ratings, as most participants reported low levels of disgust. Second, the nature of disgust was not evaluated. Future work should investigate the content of disgust among people who have experienced trauma, as disgust can be directed toward others (e.g., the perpetrator of a sexual assault) and toward aspects of the self or one's own behavior (Badour et al., 2014; Sonnier et al., 2019). To measure these aspects of disgust, more comprehensive measures of trauma‐related disgust must be developed. Third, we did not differentiate intrusive trauma thoughts from deliberate, ruminative thinking, leaving us unable to determine the unique effects of these different types of trauma‐focused thoughts. Fourth, there could have been reactivity to the EMA study design such that completing surveys up to 17 times a day may have unintentionally served as trauma reminders that may not have been present otherwise. In turn, this could have invited increases in trauma‐related experiences that were triggered by the study rather than experiences encountered in daily life. Participants were required to enter the lab each day of participation, which is another deviation from their ecologically valid context of living. Fifth, an a priori power analysis was not conducted. Thus, it is possible that the study was underpowered to detect null results. Finally, our sample was predominantly female (75.0%), included a fairly high percentage of participants who endorsed sexual trauma as their index traumatic event (38.5%), and excluded participants based on age and a number of other criteria that may limit the generalizability of these results.

Disgust is a common emotional reaction that can occur both during and following a traumatic event. In the present study, exactly 50% of participants endorsed feelings of disgust one or more times in the study despite only 3 days of data collection. Disgust was associated with trauma reminders encountered in the daily lives of trauma‐exposed community members. Overall, individuals with more severe PTSD symptoms endorsed high levels of disgust on average compared to those with less severe symptoms. The association between trauma reminders and disgust was moderated by PTSD symptom severity for all trauma reminder types except flashbacks; however, trauma type (sexual vs. nonsexual) did not moderate any effects. The present study replicated previously established associations between trauma reminders and disgust but did so in more ecologically valid contexts. It is critical that the trauma researchers and treatment providers acknowledge the unique role that disgust might play in posttraumatic emotions and symptoms. It is important to understand these associations so trauma treatment can better address emotional reactivity to specific trauma reminder types. Future research should explore the range of trauma reminder types survivors experience in daily living above and beyond traditionally studied external reminders evoked through imaginal exposure.

## AUTHOR NOTE

This work was funded by a National Institute of Mental Health grant awarded to Blair E. Wisco (R15MH114142). We have no conflicts of interest to disclose.

## OPEN PRACTICES STATEMENT

The study reported in this article was not formally preregistered. Neither the data nor the materials have been made available on a permanent third‐party archive; requests for the data or materials should be sent via email to the lead author at msnester@uncg.edu.

## Supporting information



Supporting Material
