# Supplementary material for: Trauma reminders and disgust: The roles of posttraumatic stress disorder symptom severity, trauma type, and reminder type
Source: J Trauma Stress. 2024 Jul 18;37(6):959–70. doi: 10.1002/jts.23076 (PMC11629848; doi:10.1002/jts.23076)
Supplement: Supplementary file 1 — Supporting Material [file JTS-37-959-s001.docx]

| **Table S1**  *Demographic and Trauma Characteristics* | | | | | |
| --- | --- | --- | --- | --- | --- |
| Variable | | Full Sample (*n* = 80) | SA Index Trauma (*n* = 31) | Non-SA Index Trauma (*n* = 49) | Difference Test |
| Demographic Characteristics | | | | | |
| Age (*M*, *SD*) | | 21.79 (4.21) | 21.87 (3.86) | 21.73 (4.45) | *t*(78) = -0.14, *p* = .889 |
| Gender (*n*, % women) | | 60 (75.00%) | 26 (83.87%) | 34 (69.39%) | χ^2^(1) = 2.12, *p* = .145 |
| Race/ethnicity | |  |  |  | χ^2^(5) = 7.52, *p* = .185 |
|  | White (non-Hispanic) | 28 (35.00%) | 14 (45.16%) | 14 (28.57%) |  |
|  | Black/African American | 23 (28.75%) | 8 (25.81%) | 15 (30.61%) |  |
|  | Hispanic/Latino | 17 (21.25%) | 8 (25.81%) | 9 (18.37%) |  |
|  | Asian/Pacific Islander | 2 (2.50%) | 0 (0.00%) | 2 (4.08%) |  |
|  | Biracial | 4 (5.00%) | 1 (3.23%) | 3 (6.12%) |  |
|  | Other/Multiracial | 6 (7.50%) | 0 (0.00%) | 6 (12.24%) |  |
| Trauma Characteristics | | | | | |
| Index Trauma (*n*, %) | |  |  |  | χ^2^(11) = 80.00, *p* < .001 |
|  | Sexual assault | 31 | 31 (100.00%) | 0 (0.00%) |  |
|  | Other uncomfortable sexual experience | 7 | 0 (0.00%) | 7 (7.78%) |  |
|  | Physical Assault | 17 | 0 (0.00%) | 17 (34.69%) |  |
|  | Natural disaster/accident/fire | 11 | 0 (0.00%) | 11 (22.45%) |  |
|  | Serious illness/injury/death/suffering | 10 | 0 (0.00%) | 10 (52.63%) |  |
|  | Combat exposure | 1 | 0 (0.00%) | 1 (2.04%) |  |
|  | Other | 3 | 0 (0.00%) | 3 (6.12%) |  |
| PTSD Symptom Severity (*M*, *SD*) | | 19.81 (12.07) | 22.90 (12.23) | 17.86 (11.67) | *t*(78) = 1.85, *p* = .068 |
| PTSD Status (*n*, % with PTSD) | | 39 (48.75%) | 18 (58.06%) | 21 (42.86%) | χ^2^(1) = 1.76, *p* = .185 |

| **Table S2**  *Study Variable Correlations* | | | | | | | | |
| --- | --- | --- | --- | --- | --- | --- | --- | --- |
| Variable | *M* (*SD*) | 1 | 2 | 3 | 4 | 5 | 6 | 7 |
| 1. Disgust | 1.13 (0.62) | 1 |  |  |  |  |  |  |
| 2. CAPS-5 | 19.81 (11.99) | .16^***^ | 1 |  |  |  |  |  |
| 3. Trauma Type | 0.39 (0.49) | -.03 | .21^***^ | 1 |  |  |  |  |
| 4. Trauma Reminder Composite | 0.28 (0.45) | .21^***^ | .22^***^ | -.11^***^ | 1 |  |  |  |
| 5. Trauma Thoughts | 0.22 (0.41) | .24^***^ | .16^***^ | -.06^**^ | .84^***^ | 1 |  |  |
| 6. Intrusive Memories | 0.16 (0.36) | .26^***^ | .19^***^ | -.05^*^ | .69^***^ | .74^***^ | 1 |  |
| 7. Flashbacks | 0.04 (0.20) | .30^***^ | .12^***^ | -.07^***^ | .34^***^ | .36^***^ | .45^***^ | 1 |
| 8. External Reminders | 0.22 (0.41) | .22^***^ | .24^***^ | -.09^***^ | .85^***^ | .66^***^ | .61^***^ | .36^***^ |
| *Note*. Correlations between continuous variables represent Pearson correlation coefficients. Correlations between a continuous variable and dichotomous variable represent Point-Biserial correlation coefficients. Correlations between two dichotomous variables represent Phi coefficients. *** *p* < .001. ** *p* < .01. * *p* < .05. | | | | | | | | |

|  | | | | | | |  |
| --- | --- | --- | --- | --- | --- | --- | --- |
| **Table S3**  *Mode Fit Indices* | | | | | | | |
|  | | Reminder Composite | Trauma Thoughts | Intrusive Memories | Flashbacks | External Reminder | |
| Trauma Reminder Only Model | | | | | | | |
|  | AIC | 3392.68 | 3282.32 | 3309.40 | 3369.24 | 3428.19 | |
|  | BIC | 3421.07 | 3310.70 | 3337.79 | 3397.63 | 3456.57 | |
| Full Model | |  |  |  |  |  | |
|  | AIC | 3382.93 | 3272.31 | 3301.22 | 3365.48 | 3419.10 | |
|  | BIC | 3434.02 | 3323.40 | 3352.31 | 3416.57 | 3470.19 | |
| *Note*. AIC = Akaike information criterion. BIC = Bayes information criterion. | | | | | | | |
